# Thermodynamics of Aryl-Dihydroxyphenyl-Thiadiazole Binding to Human Hsp90

**DOI:** 10.1371/journal.pone.0036899

**Published:** 2012-05-24

**Authors:** Egidijus Kazlauskas, Vilma Petrikaitė, Vilma Michailovienė, Jurgita Revuckienė, Jurgita Matulienė, Leonas Grinius, Daumantas Matulis

**Affiliations:** 1 Department of Biothermodynamics and Drug Design, Vilnius University Institute of Biotechnology, Vilnius, Lithuania; 2 Department of Drug Chemistry, Faculty of Pharmacy, Lithuanian University of Health Sciences, Kaunas, Lithuania; Institute of Enzymology of the Hungarian Academy of Science, Hungary

## Abstract

The design of specific inhibitors against the Hsp90 chaperone and other enzyme relies on the detailed and correct understanding of both the thermodynamics of inhibitor binding and the structural features of the protein-inhibitor complex. Here we present a detailed thermodynamic study of binding of aryl-dihydroxyphenyl-thiadiazole inhibitor series to recombinant human Hsp90 alpha isozyme. The inhibitors are highly potent, with the intrinsic *K_d_* approximately equal to 1 nM as determined by isothermal titration calorimetry (ITC) and thermal shift assay (TSA). Dissection of protonation contributions yielded the intrinsic thermodynamic parameters of binding, such as enthalpy, entropy, Gibbs free energy, and the heat capacity. The differences in binding thermodynamic parameters between the series of inhibitors revealed contributions of the functional groups, thus providing insight into molecular reasons for improved or diminished binding efficiency. The inhibitor binding to Hsp90 alpha primarily depended on a large favorable enthalpic contribution combined with the smaller favorable entropic contribution, thus suggesting that their binding was both enthalpically and entropically optimized. The enthalpy-entropy compensation phenomenon was highly evident when comparing the inhibitor binding enthalpies and entropies. This study illustrates how detailed thermodynamic analysis helps to understand energetic reasons for the binding efficiency and develop more potent inhibitors that could be applied for therapeutic use as Hsp90 inhibitors.

## Introduction

Heat shock protein 90 (Hsp90) is a component of the cellular chaperone machinery [1], [2]. There are a number of recent developments in the understanding of the interesting and complex mechanism of Hsp90 action [3]–[9]. Hsp90 is overexpressed in cancer cells and Hsp90 inhibitors have shown selectivity for cancer cells. Therefore, small-molecule inhibitors are being developed as anticancer therapeutics [10]–[15].

Two groups of natural product inhibitors of Hsp90, based on geldanamycin and radicicol have been discovered that bind to the N-terminal domain ATP-binding pocket. Both natural compounds have been used as leads to develop compounds with desired pharmaceutical properties such as increased potency and reduced toxicity [1], [13].

**Figure 1:**
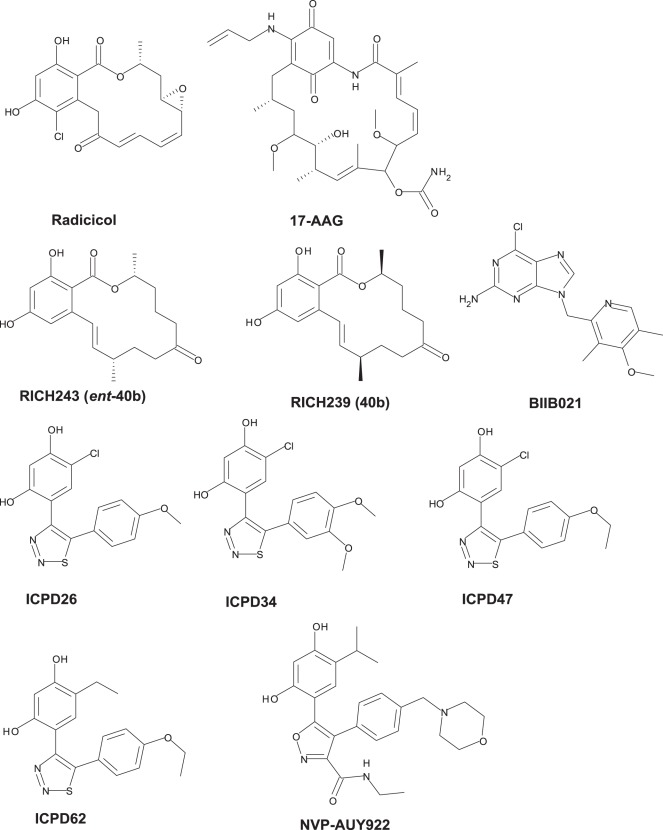
Chemical structures of selected natural and synthetic Hsp90 inhibitors. ICPD series of compounds are the subject of this study.

Experience with the natural products generated interest in alternative chemotypes, and the first synthetic inhibitors that bind the ATP-binding site at the NH_2_ terminus of Hsp90 have been designed based on a purine scaffold [16], [17]. Based on discovery of the novel synthetic 3,4-diarylpyrazole derivative of resorcinol-type Hsp90 inhibitor by high-throughput screening [18], a series of active analogues of both diarylpyrazole [19] and diarylisoxazole inhibitors [13], [20] have been generated by structure-based design. Several groups have discovered and successfully advanced to clinics new Hsp90 inhibitors. For instance, new inhibitors have been designed based on benzamide [21], on 2-aminothieno[2,3-d]pyrimidine [20] and on dihydroxyphenylisoindoline [22] scaffolds. Here we study the aryl-dihydroxyphenyl-thiadiazole inhibitor [23]–[25] binding to Hsp90. Their chemical structures together with other selected Hsp90 inhibitors from the literature are shown in Figure 1.

Despite these achievements, full thermodynamic description of the ligand binding to Hsp90 is rather fragmented despite its importance for structure-based drug development [26], [27]. The enthalpy and heat capacity of binding correlate with structural parameters such as hydrogen bond formation and hydrophobic contacts more closely than the Gibbs free energy. As the ligand binding affinity is a combined function of the binding enthalpy and the binding entropy, an improved affinity could result when any or both terms are designed to contribute more favorably to binding [28]–[30].

To characterize thermodynamic parameters of the binding of new resorcinol derivatives to the N-terminal domain of human Hsp90, we used two independent methods, ITC and thermal shift assay [31] (TSA), also known as differential scanning fluorimetry [32] and ThermoFluor® [33]. The ITC fully characterizes the thermodynamics of the binding reaction, including the *K_d_*, as well as enthalpy, entropy and heat capacity of binding [26], [27], [30], [34]. ITC accuracy of direct determination of the observed binding constant *K_b_obs_* is low if the ligand binding is too tight, while the observed enthalpy can be determined with high precision and its value can be used for calculation of a pK_b_ value [35], [36]. On the other hand, precise determination of observable binding constants using the TSA is possible for any noncovalent ligand binding to protein, even for tight ligand binding, independent of whether the ligand stabilizes or destabilizes the protein upon binding [37], [38]. Therefore, the ITC and the TSA methods complement each other for increased precision of the measurements [39].

The binding of ligands to proteins show some degree of pH dependence, reflecting the linkage between the binding of ligand and the binding of protons [35], [36], [40]. By performing experiments as a function of pH in buffers with varying ionization enthalpy, the p*K_a_* values of the group(s) responsible for the proton linkage in the free and liganded states can be determined together with the protonation enthalpy for this group in these states together with intrinsic energetic parameters of the binding.

## Results

### Isothermal Titration Calorimetry (ITC) of ICPD Compound Binding to Hsp90

**Figure 2:**
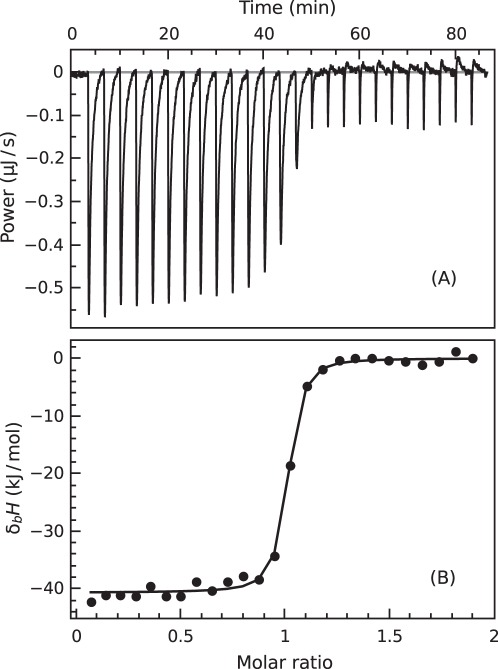
Isothermal titration calorimetry data of ICPD47 binding to Hsp90αN at pH 7.0, 37°C. Panel A shows raw data curve and Panel B shows the fitted integrated ITC data curve. The stoichiometry of binding is approximately equal to one inhibitor molecule per protein molecule.

**Figure 3:**
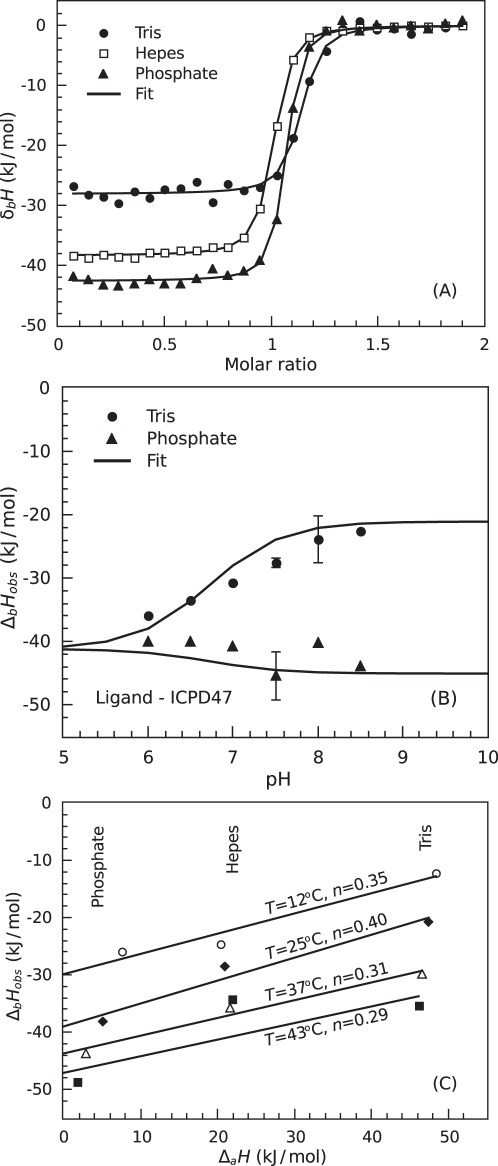
The pH and buffer effects of ICPD inhibitor binding to Hsp90. Panel A. ICPD47 binding to Hsp90αN by ITC in three buffers: ▴ – sodium phosphate, □ – Hepes, and • – Tris chloride buffer, at pH 7.5 and 37°C. Note that the observed enthalpy of binding is significantly more exothermic in phosphate than in Tris buffer. This indicates the presence of a binding-linked protonation event. Panel B. The observed enthalpies of ICPD47 binding to Hsp90αN as a function of pH in two buffers: ▴ – phosphate, and • – Tris. The data points are the enthalpies that were observed experimentally using ITC, and the lines are fitted to the single binding-linked protonation model, Eqs. (1–4). Observed binding enthalpies differ in the two buffers at higher pH values because of a linked protonation event, and because Tris and phosphate have different deprotonation enthalpies. The intrinsic binding enthalpy is the point at which the two curves meet at low pH values (∼−40 kJ/mol). Panel C. The observed enthalpies of ICPD47 binding to Hsp90αN as a function of the buffer deprotonation enthalpy at various temperatures: ○ – 12°C, ⧫ – 25°C, ▵ – 37°C, and ▪ – 43°C. The data points are the experimentally observed enthalpies, and the trendlines are linear fits. Their slopes are equal to the binding-linked protonation events (*n*). There was little change of *n* as a function of temperature. The zero intercepts of the lines are equal to the buffer-independent binding enthalpies. The enthalpies are not intrinsic binding enthalpies because they contain the heats of protonation of protein/ligand functional groups.

The energetics of ICPD compound binding to Hsp90 was measured using ITC. Figure 2 shows a representative raw data titration of the Hsp90 N-terminal domain (Hsp90αN) with ICPD47 in 50 mM sodium phosphate buffer, pH 7.0, in 100 mM NaCl, at 37°C. The binding reaction was strongly exothermic and exhibited steep slope of the ITC curve indicating tight binding reaction. All tested ICPD compounds bound to all tested Hsp90 constructs with the stoichiometry of one inhibitor molecule per one protein molecule within the error of ITC measurements (Figures 2 and 3A). There was essentially no difference in the binding curve observed with the Hsp90αN and the full length Hsp90 protein (Hsp90αF). Therefore, the binding reaction to the N-terminal domain closely resembles the binding reaction to the Hsp90αF. Table 1 lists representative ITC data for the series of inhibitor binding to Hsp90αN and Hsp90αF at a wide range of pH, buffer, and temperature.

**Table 1:** Representative ITC data for the binding of ICPD compounds to recombinant human Hsp90 protein constructs as a function of pH, temperature, and buffer ionization enthalpy.

Compound	*T*, °C	pH	Buffer	*K_d_*, nM	Δ*_b_H_obs_*, kJ×mol^−1^
Hsp90αN					
ICPD 47	37	6	Pi^a^	7.30	−40.1
	37	6.5	Pi	4.13	−39.9
	37	7	Pi	6.80	−40.7
	37	7.5	Pi	6.17	−42.6
	37	8	Pi	14.5	−40.1
	37	8.5	Pi	45. 5	−43.9
	37	9	Pi	84.8	−64.8
	37	9.5	Pi	111	−50.9
	37	10	Pi	400	−49.7
	37	10.5	Pi	699	−51.5
	37	11	Pi	1560	−26.5
	37	11.5	Pi	9620	−34.4
	37	6	Tris	9.35	−35.9
	37	6.5	Tris	2.84	−33.4
	37	7	Tris	10.9	−30.8
	37	7.5	Tris	8.20	−27.1
	37	8	Tris	57.5	−26.5
	37	8.5	Tris	86.2	−22.6
	37	9	Tris	88.5	−26.1
	37	9.5	Tris	550	−29.7
	37	10	Tris	510	−23.1
	12	7.5	Hepes	5.0	−29.1
	25	7.5	Hepes	8.0	−28.6
	37	7.5	Hepes	13.9	−44.4
	43	7.5	Hepes	13.3	−44.1
	12	7.5	Pi	7.58	−33.7
	25	7.5	Pi	7.19	−41.1
	37	7.5	Pi	10.8	−51.8
	43	7.5	Pi	21.7	−65.4
	12	7.5	Tris	8.13	−14.7
	25	7.5	Tris	6.71	−25.0
	37	7.5	Tris	10.0	−32.9
	43	7.5	Tris	8.23	−47.8
ICPD 62	7	7.5	Hepes	0.22	−12.4
	12	7.5	Hepes	0.52	−18.3
	25	7.5	Hepes	0.27	−21.2
	37	7.5	Hepes	9.09	−25.5
	43	7.5	Hepes	3.28	−34.8
	7	7.5	Pi	0.31	−11.9
	12	7.5	Pi	3.89	−14.1
	25	7.5	Pi	0.19	−19.4
	37	7.5	Pi	5.56	−18.5
	43	7.5	Pi	0.85	−36.6
	7	7.5	Tris	0.19	−10.8
	12	7.5	Tris	0.33	−14.2
	25	7.5	Tris	1.29	−17.0
	37	7.5	Tris	14.6	−28.7
	43	7.5	Tris	14.9	−34.2
ICPD 34	7	7.5	Hepes	2.75	−23.5
	12	7.5	Hepes	6.17	−25.7
	25	7.5	Hepes	17.7	−31.6
	37	7.5	Hepes	98.0	−49.5
	43	7.5	Hepes	53.5	−44.4
	7	7.5	Pi	9.07	−31.2
	12	7.5	Pi	5.03	−28.8
	25	7.5	Pi	12.7	−38.4
	37	7.5	Pi	56.2	−49.5
	43	7.5	Pi	55.0	−53.3
	7	7.5	Tris	1.59	−11.7
	12	7.5	Tris	4.13	−14.3
	25	7.5	Tris	12.6	−21.0
	37	7.5	Tris	41.0	−28.9
	43	7.5	Tris	70.4	−35.7
ICPD 26	12	7.5	Hepes	3.13	−28.4
	25	7.5	Hepes	3.65	−36.9
	37	7.5	Hepes	12.6	−42.9
	43	7.5	Hepes	17.5	−53.2
	12	7.5	Pi	2.59	−50.1
	25	7.5	Pi	7.60	−42.0
	37	7.5	Pi	16.2	−48.4
	43	7.5	Pi	18.0	−61.6
	12	7.5	Tris	6.17	−16.2
	25	7.5	Tris	6.54	−27.0
	37	7.5	Tris	8.36	−36.6
	43	7.5	Tris	6.58	−44.7
**Hsp90αF**					
ICPD 47	12	7.5	Hepes	6.06	−32.6
	25	7.5	Hepes	6.17	−35.4
	37	7.5	Hepes	8.40	−39.0
	43	7.5	Hepes	7.46	−44.1
	12	7.5	Pi	4.06	−33.7
	25	7.5	Pi	4.81	−42.1
	37	7.5	Pi	8.37	−50.2
	43	7.5	Pi	15.6	−60.6
	12	7.5	Tris	1.29	−18.7
	25	7.5	Tris	3.14	−25.7
	37	7.5	Tris	3.50	−35.9
	43	7.5	Tris	8.35	−40.5

Repeated experiments at identical conditions in different series are shown to illustrate the level of experimental error involved in these results.

a Pi is sodium phosphate buffer. The standard deviations were about 3 kJ×mol^−1^ for the enthalpy and up to 1.6 fold for the *K_d_*, especially when the binding was too tight to be measured accurately using ITC.

The observed enthalpy of binding was highly dependent on the buffer used in the ITC experiment (Figure 3A). For example, the observed enthalpy was approximately −28 kJ/mol in Tris buffer and −42 kJ/mol in phosphate buffer. These buffers have significantly different enthalpies of deprotonation. Therefore, there are one or many binding-linked (de)protonation events. Furthermore, analysis of observed enthalpies for ICPD47 as a function of pH (Figure 3B) shows a typical pH dependence indicating a single protonation event linked to inhibitor binding. However, the ICPD62 did not exhibit such strong protonation event near pH7 because the p*K_a_* was significantly higher as explained later.

The observed enthalpies of binding plotted as a function of buffer deprotonation enthalpy (Δ_a_ *H*, Figure 3C) yielded the number of protons being uptaken from solution upon inhibitor binding at various temperatures. The proton number dependence on temperature (line slope in Figure 3C) was negligible and it was primarily dependent on solution pH.

The *c* value (*c*  =  *C*×*K_b_*, *C* is the molar concentration of the protein, and *K_b_* is the binding constant, when the binding stoichiometry is 1∶1) for an ITC titration must be between 1 and 1000 (or more narrowly 5 to 500) to yield the curve where the slope is not too steep or to shallow for accurate fitting [41], [42]. In order not to exceed the *c*  = 1000, at our experimental conditions of 6 µM protein in the calorimeter cell, we can only accurately measure the observed binding constants up to 1.6×10^8^ M^−1^(or 8×10^7^ M^−1^) The binding is tighter at lower pH and exceeds the limit of accurate determination of binding strength. Therefore, an additional method could increase the precision and verify the binding constants.

### Thermal Shift Assay (TSA) of ICPD Compound Binding to Hsp90

The thermal shift assay measures the protein-ligand binding constant by determining the increase in the melting temperature of the protein that is caused by the ligand [37], [39], [43]. Protein unfolding is monitored by following the fluorescence of an extrinsic probe (such as 1,8-anilinonaphtalene sulfonate, ANS) upon increasing the temperature of the protein solution at a constant heating rate. Soluble single-domain globular proteins melt with a single transition that is affected by ligands. Full-length Hsp90 yields a complicated multi-domain melting transition profile and its analysis is not straightforward. However, the N-terminal domain of Hsp90 yields a single transition that can be used to study inhibitor binding to Hsp90.

**Figure 4:**
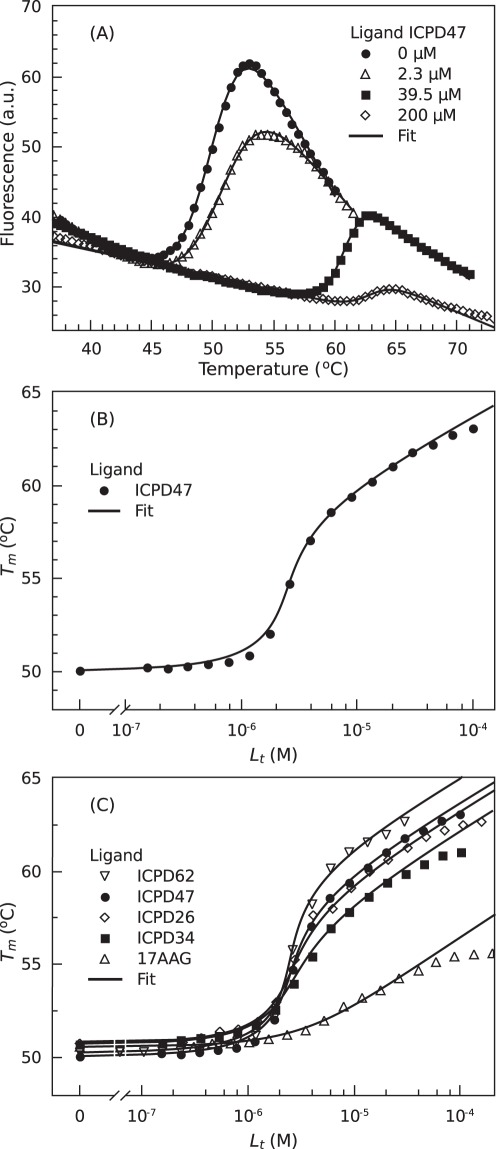
ICPD compound binding to Hsp90 by thermal shift assay (TSA). Panel A. The raw fluorescence TSA data of ICPD47 binding to Hsp90αN at pH 7.5. The Hsp90αN thermal denaturation transitions (*T_m_*) were increasingly shifted upwards as the concentrations of ICPD47 increased: • – 0 µM, ▵ – 2.34 µM, ▪ – 39.5 µM, and ⋄ – 200 µM. The lines are fits according to Eq. (5). Panel B. The melting temperatures (*T_m_*) from data as in Panel A plotted as a function of added ICPD47 concentration. The line is a fit according to Eq. (6). Panel C. The *T_m_* TSA data as a function of the concentration of various added compounds: ▿ – ICPD62, • – ICPD47, ⋄ – ICPD26, ▪ – ICPD34, and ▵ – 17AAG. The data points are obtained from the raw data as in Panel A, and the lines are fitted according to the model, Eq. (6). Note that ICPD62 shifts the *T_m_* to the greatest extent and thus the observed *K_b_obs_* is largest (but not the intrinsic *K_b_*).

Figure 4A shows ANS fluorescence curves as a function of temperature in the absence and presence of various ICPD47 inhibitor concentrations. The concentration of Hsp90αN was 10 µM. With no inhibitor added, there is a steep increase in fluorescence observed at approximately 50°C (pH 7.0). This increase is due to protein unfolding that exposes hydrophobic surfaces where ANS can bind and be excluded from quenching by the aqueous environment [44]. ANS actually binds through a combination of hydrophobic and ionic interactions but the ion pairs between the ANS sulfonate groups and the protein amino groups are not visible by fluorescence [45], [46]. Addition of ICPD47 shifted the transition midpoint towards higher temperatures because the ligand stabilized the protein upon binding.

Protein melting temperatures (*T_m_*) at various inhibitor concentrations were determined by fitting the protein melting curves as described in the Materials and Methods section. The resultant transition midpoints were plotted as a function of added ligand concentration yielding the ligand-dosing curves (Figure 4B). Datapoints make a sigmoidal shape curve where the steepest increase is observed at a ligand concentration which is similar but slightly lower than the protein concentration (10 µM). The observed binding constant is obtained by fitting the datapoints using Eq. (6).

Figure 4C compares TSA ligand-dosing curves for ICPD compounds and 17-AAG. Similar curves were previously published for radicicol [47]. The four strongly-binding ICPD compounds exhibited a large *T_m_* shift of more than 10°C. Exact temperature shift is strongly concentration-dependent. The compounds ranked in this order of *T_m_* shift and the observed binding constant (*K_b_*): ICPD62> ICPD47> ICPD26> ICPD34. The observed binding constants were equal to 1.7×10^8^, 8.0×10^7^, 3.8×10^7^, and 1.2×10^7^ M^−1^, respectively. The 17-AAG exhibited a significantly weaker *T_m_* shift with the *K_b_*  = 6.0×10^5^ M^−1^.

### Interpretation of Binding-linked Protonation Events

**Figure 5:**
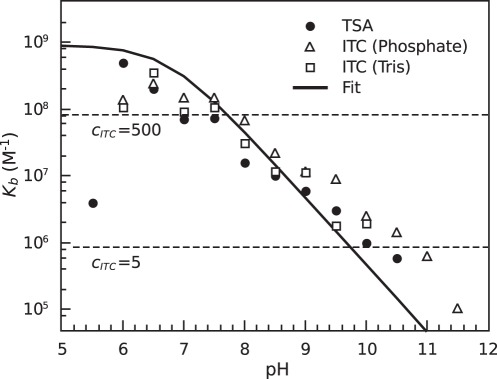
The observed binding constant (*K_b_obs_*) dependence on pH. The observed binding constants of the interaction of ICPD47 with Hsp90αN obtained using three experimental approaches: ▵ – ITC in phosphate buffer, □ – ITC in Tris buffer, and • – TSA, all at 37°C. There is a clear decrease in the binding affinity at higher pH. The line is fitted according to Eq. (1) for a linked protonation event using p*K_a_^f^*  = 6.72. Note, that the ITC data does not provide an accurate measure of the binding constant at pH values below 8.0 because the binding is too tight, as shown by the dashed line drawn for the ITC *c* factor equal to 500.

ICPD compound interaction with Hsp90 was increasingly weaker with increasing pH. The observed binding constant, as determined both by ITC and TSA, diminished by about 1 order of magnitude with each pH unit (Figure 5). This decrease was attributed to a binding-linked protonation event. The analysis of binding-linked protonation events was done as previously described [35], [40] and applied to Hsp90 [47]–[49].

The pH at which the decrease in observed *K_b_* is monitored was not the same for various compounds. ICPD62 exhibited the decrease in *K_b_obs_* at greater pH than ICPD47. Compounds ICPD62 and ICPD47 have significantly different ionization p*K_a_*s, equal to approximately 8.45 and 6.72, respectively. These p*K_a_*s could be attributed to the hydroxyl groups adjacent to chlorine in ICPD47 and to ethyl group in ICPD62. Their values are so different because of different electron withdrawing capacity by the chlorine and ethyl groups in the *ortho* position from the hydroxyl group.

**Figure 6:**
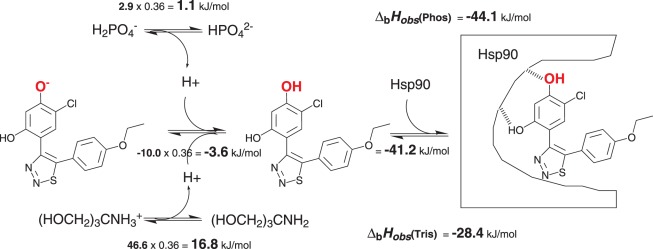
Enthalpic contributions of the binding-linked reactions are shown, including, the protonation of the compound (ICPD47 ) hydroxy group (bold and red), buffer ionization, and the intrinsic enthalpy of binding into the experimentally observed values.

Figure 6 shows the linked protonation event, the protonation of the hydroxyl group at high pH, upon binding to Hsp90. The enthalpic contributions to all linked reactions, including buffer deprotonation and compound protonation are shown. These linked reactions make large enthalpic contributions that should be accounted for in order to dissect the intrinsic binding parameters of each ligand to the protein molecule. The enthalpy of protonation of the hydroxyl group of ICPD47, equal to −10.0 kJ/mol, was obtained by fitting all titration data and has the uncertainty of approximately ±5 kJ/mol.

**Figure 7:**
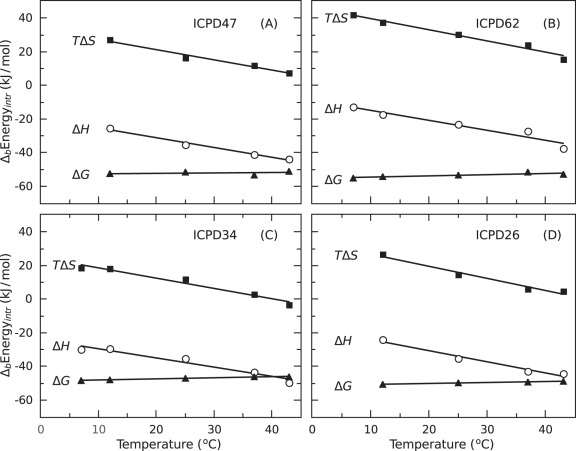
The intrinsic binding thermodynamic parameters ( ○ **– enthalpies, ▪ – entropies, and ▴ – Gibbs free energies) of ICPD47 (Panel A), ICPD62 (Panel B), ICPD34 (Panel C), and ICPD26 (Panel D) to Hsp90 plotted as a function of temperature.** The intrinsic binding parameters are listed in Table 2.

**Table 2:** The intrinsic thermodynamic parameters of ICPD compound binding to Hsp90 at 37°C.

Protein	Compound	*K_b_*, nM^−1^	*K_d_*, nM	Δ*_b_H_intr_*, kJ×mol^−1^	Δ*_b_G_intr_*, kJ×mol^−1^	*T*Δ*_b_S_intr_*, kJ×mol^−1^	Δ*_b_S_intr_*, J×mol^−1^×K^−1^	Δ*_b_C_p_*, J×mol^−1^×K^−1^
Hsp90αN	ICPD 47	9×10^8^	1.1	−41.2	−53.2	12.0	38.6	−570
Hsp90αF	ICPD 47	9×10^8^	1.1	−46.6	−53.2	6.6	21.2	−570
Hsp90αN	ICPD 62	5×10^8^	2.0	−27.5	−51.7	24.2	77.9	−590
Hsp90αN	ICPD 34	6×10^7^	16.7	−43.3	−46.2	2.9	9.3	−540
Hsp90αN	ICPD 26	2×10^8^	5.0	−43.1	−49.3	6.2	20.0	−660
Uncertainty		±1.6×	±1.6×	±3.0	±2.6	±4.0	±14	±120

### Intrinsic Binding of ICPD Compounds to Hsp90 Protein

Various binding-linked reactions, usually protonation, make the analysis of the binding reaction more complex. However, it is necessary to be dissected in order to determine the intrinsic binding parameters. Only the intrinsic binding parameters could be used to correlate with the structural features of the protein-ligand complex. In this case, intrinsic parameters refer to the binding of electrically neutral, protonated ICPD compound. Intrinsic thermodynamic parameters are shown in Figure 7 as a function of temperature and listed in Table 2.

**Figure 8:**
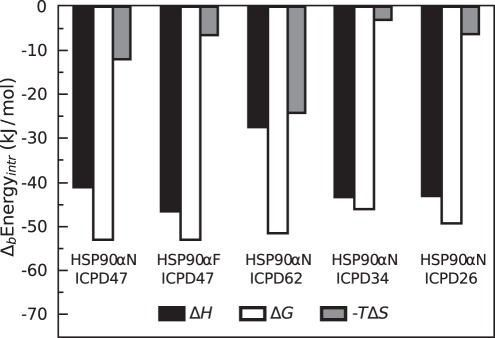
Bar chart comparing relative contributions of intrinsic enthalpies and intrinsic entropies to the Gibbs free energies of binding of ICPD compound to Hsp90 at 37°C. Note, that with the partial exception of ICPD62, the compounds bound with major exothermic enthalpy contribution and minor favorable entropy contribution. Favorable contributions of both components make the compounds such potent binders.

Dissection of the inhibitor binding thermodynamics into their enthalpic and entropic contributions shows that ICPD-Hsp90 binding is driven by enthalpy and by a lesser extent –entropy (Figure 8). The entropic component accounts for approximately only 20% of the energy favoring binding. About 80% of the binding energy comes from the favorable enthalpic contribution. However, ICPD62 binding was somewhat different with enthalpic and entropic contributions to binding being nearly equal. The Gibbs free energies of binding to the full-length protein and the N-terminal domain were similar but enthalpically more favorable to Hsp90aF by 5.4 kJ/mol while entropically less favorable by the same amount.

The slopes of the intrinsic binding enthalpies as a function of temperature give the intrinsic heat capacities of ICPD compound binding to Hsp90. The heat capacity of all tested ICPD compound binding to Hsp90αN is within the range of −540 to −660 J×mol^−1^×K^−1^. The negative heat capacity is a signature of hydrophobic binding reactions, and the value is approximately of the expected magnitude for an ICPD molecule. The heat capacities of binding slightly correlate with the burial of hydrophobic surfaces upon binding [50], but these were not calculated in the present study.

### Compound Functional Group Contributions to the Binding Thermodynamic Parameters

**Figure 9:**
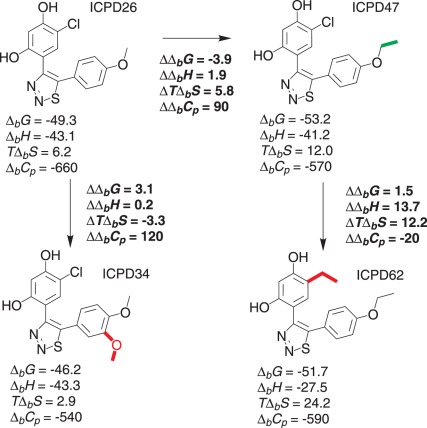
Functional group contributions to the intrinsic Gibbs free energies (Δ*_b_G*), enthalpies (Δ*_b_H*), entropies (*T*Δ*_b_S*), and the heat capacities (Δ*_b_C_p_*) of binding shown as differences (Δ) between the binding parameters. Groups favoring binding Δ*_b_G* are shown in bold green, while unfavoring binding – in bold red. Numbers are energies in kJ/mol except for heat capacity in J×mol^−1^×K^−1^. It should be noted that the uncertainties of the values are ±2.6 for the Gibbs free energies, ±3.0 for enthalpies, ±4.0 for entropies, and ±120 for the heat capacities. Therefore, the differences for some contributions are within the uncertainty margins.

When all intrinsic thermodynamic parameters of compound binding to Hsp90 are determined, it is important to calculate the differences and correlate them with the differences in functional groups. Figure 9 shows the differences in binding parameters as ΔΔ*_b_X*. For example, the addition of methylene group going from ICPD26 to ICPD47 (horizontal arrow) contributes favorably −3.9 kJ/mol of the binding Gibbs free energy. Therefore, compound ICPD47 binds more strongly to Hsp90 than ICPD26. The methylene group is shown in green as favorably contributing to the binding Gibbs free energy. However, its contribution to the enthalpy of binding is slightly unfavorable.

The change of chlorine atom to the ethyl group (right vertical arrow, going from ICPD47 to ICPD62) yielded an unfavorable contribution both to the Gibbs free energy and enthalpy of binding. Therefore, the ethyl group is shown in red as contributing unfavorably to Δ*_b_G*.

Note that the observed binding constant as determined by TSA was slightly greater for ICPD62 than ICPD47 (Figure 4). The observed binding thermodynamic parameters do not correlate with the intrinsic parameters in this case. Therefore, without correct determination of the intrinsic thermodynamic parameters, one would make erroneous conclusions about the contributions of the functional groups to the binding energetics.

## Discussion

Despite the difficulties involved and the large number of ITC titrations required to carry out the full thermodynamic proton linkage analysis, it is important to dissect the linkage to obtain the intrinsic binding parameters of any ligand. The binding of inhibitor to Hsp90 is a good example of a single binding-linked protonation event for which the thermodynamic characterization of binding would yield misleading results without the linkage analysis.

The data listed in Table 1 illustrates the error margin for the ITC measurements. For example, the enthalpies of ICPD47 binding to Hsp90aN in Tris buffer at 37°C and pH7.5 in two listed repeats were −27.1 and −32.9 kJ/mol. The uncertainty of the resultant enthalpy is about ±3.0 kJ/mol. Figure 3B and 3C also illustrate the margin of the data scatter.

The small differences in thermodynamic parameters of binding various compounds make it difficult to put together a reliable functional group additivity scheme. However, some trends for the thermodynamics of binding can be visualized in Figure 9. Beginning with ICPD26, the addition of methyl group (ICPD47) made the binding tighter by −3.9 kJ/mol, but enthalpically less favorable by +1.9 kJ/mol. Improved binding was fully due to an increased entropic contribution. However, addition of a second methoxy group (ICPD26 → ICPD34) diminished the binding potency by about +3.1 kJ/mol. Since the enthalpies of binding were essentially identical, the loss of potency was due to the loss of entropy. Replacement of chlorine atom with the ethyl group (ICPD47 → ICPD62) slightly diminished the potency by +1.5 kJ/mol. However, the loss of favorable enthalpic contribution (+13.7 kJ/mol) and the gain of entropic favorable contribution (+12.2 kJ/mol) was significant.

Truncating the protein (comparing Hsp90aF and Hsp90aN) did not affect the binding constant significantly as measured by ITC. However, it should be kept in mind that the binding reaction is too tight to be measured by ITC since the *c* factor is >500. Therefore, more precise data for *K_b_obs_* determination was obtained by TSA. However, it was more difficult to determine the *K_b_obs_* for the full protein due to the complicated unfolding temperature curve. Therefore, the *K_b_obs_* data are based primarily on ITC data comparison at pH values where reliable ITC data could be obtained (i.e., pH 8.0–9.0).

The dissection of linked protonation events was somewhat ambiguous. The difference between the enthalpies of binding in phosphate and Tris buffers did not reach the *n* value that would be equal to one proton being uptaken from the solution. At high pH, the *n* was equal to approximately 0.6 to 0.7. Therefore, additional compensating protonation events could be occurring. One possibility is that the Asp93 residue protonation may be also linked to the binding reaction. However, such a possibility is less likely because the two protonation events (Asp93 and OH of the inhibitor) would sum up to make the *net* number *n*  = 2. Alternatively, the Asp93 residue may be the sole protonation-linked to binding instead of the hydroxyl group of the inhibitor. However, such explanation is not supported by the fact that the p*K_a_* difference between ICPD47 and ICPD62 closely correlates with the observed binding thermodynamics as shown in Figure 6.

The structural arrangement of ICPD inhibitors in the active site of Hsp90 may be inferred from comparison with crystal structures of similar compounds bound to Hsp90 that are available in PDB. ICPD inhibitors bear resorcinol group and should bind similarly to the naturally occurring compound radicicol (PDB ID 1bgq, energetics analyzed in [47]) or inhibitors such as 4-chloro-6-(4-piperazin-1-yl-1h-pyrazol-3-yl)-benzene-1,2-diol (PDB ID 2ccs [51]). One hydroxyl group of the compound makes direct hydrogen bond with Asp93 and the nitrogen of the pyrazole ring makes the hydrogen bond with the carbonyl oxygen of Gly97. The chlorine atom makes hydrophobic contact with Phe138. There are also other hydrophobic contacts. However, it also appears that several contacts mediated by water molecules to the protein molecule make significant contribution to the energy of binding. Unfortunately, without crystal structures, the comparison between the ICPD compounds is not feasible.

This study describes the thermodynamics of ICPD compound binding to Hsp90. These compounds are enthalpically optimized single-digit nanomolar binders of Hsp90. The compounds are potent killers of osteosarcoma and HeLa cancer cells, thus, the compounds may be developed into therapeutically useful Hsp90 inhibitors.

## Materials and Methods

### Materials

5-Aryl-4-(5-substituted-2,4-dihydroxyphenyl)-1,2,3-thiadiazole compounds (abbreviated ICPD) were synthesized and purified as previously described [25]. Compound stocks were prepared by dissolving them in DMSO at 20 mM concentration. The concentration of compounds for calorimetry experiments was determined spectrophotometrically.

### Hsp90 Constructs

Hsp90αN – the N-terminal domain of alpha Hsp90 (corresponding to amino acids 1-241) was inserted into the pET21b vector (Novagen, Madison, WI, USA) using NdeI and BamHI restriction sites. Hsp90αF – Full length alpha Hsp90 was cloned and purified as previously described [25].

### Protein Expression and Purification

Hsp90αN protein was expressed in the *Escherichia coli* strain BL21 (DE3). Bacterial cultures were grown until A_550_ of 0.5–0.8 was reached and the expression was induced by the addition of 1 mM IPTG. Cells were lysed by sonication. Soluble protein was purified using an anion exchange chromatography column containing DEAE- sepharose (GE-Healthcare Bio-Sciences AB SE-751 84, Uppsala) followed by size exclusion chromatography column containing Sephacryl S-200 (Pharmacia AB Laboratory Separation Division, Uppsala Sweden).

His_6_-tagged Hsp90αF protein was expressed in the *Escherichia coli* strain BL21 (DE3). Protein was purified using a Ni-IDA affinity column, followed by an anion exchange chromatography column. SDS-PAGE analysis of both Hsp90αN and Hsp90αF showed the protein purity to be higher than 98%. Protein concentrations were determined by UV-VIS spectrophotometry and confirmed by standard Bradford method.

### Isothermal Titration Calorimetry

The protein solutions (2–6 µM) were loaded into VP-ITC isothermal titration calorimeter (Microcal, Inc.) cell (active cell volume of 1.4 ml). The solution was titrated with 20–60 µM ligand solution, 25 injections, at 3–4 minute intervals each, using 250 µl titration syringe. The stirring speed was set to 260 rpm. Experiments were carried out at constant temperature in 7–43 °C temperature range. The ligand solutions were prepared in the same buffer as the protein solutions, and with the same concentration of DMSO (usually 1%). Most titration experiments were repeated at least twice. Some experiments, such as titrations at intermediate pH values, were performed once. Experiments at standard and limiting conditions of pH, temperatures, were repeated at least three times.

### Analysis of the Linked Protonation Events

A theoretical treatment demonstrating the use of ITC measurements to dissect proton linkage from ligand binding was given by Murphy and coworkers [35], [40], [52] with the application to Hsp90 in [47]. If a protein has a single ligand-binding site and a single proton uptake is linked to the binding process (i.e., a proton is taken from the buffer solution), then there are four linked processes described by the thermodynamic parameters, namely, ligand binding to the unprotonated and protonated protein form and proton binding to unliganded and ligand-bound protein form. When ligand binding affects and shifts the *pK_a_* of any ionizable group on a protein molecule, the binding and protonation events are linked. All thermodynamic parameters, such as the Gibbs free energies (or binding constants), enthalpies, entropies, and the heat capacities, are additive. As it takes energy to shift the *pK_a_*, the binding constant of a ligand would be diminished at the pH at which the proton needs to be taken or given to the buffer. If ligand binding is linked to the binding of a single proton, then the observed binding constant (*K_obs_*) and the intrinsic binding constant (*K_intr_*) are related by:
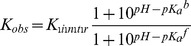
(1)


*K_a_^b^* and *K_a_^f^* are the proton dissociation constants from the liganded and unliganded protein, respectively. The change in the number of protons bound by the protein upon binding of the ligand (*n*) is the difference between the fractional saturation of protons in the free and liganded protein:
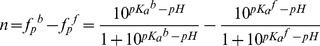
(2)


The value of *n* can be determined by ITC because it contributes to the observed binding enthalpy (Δ*_b_H_obs_*):
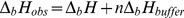
(3)


Δ*_b_H* is the enthalpy that would be measured in a buffer that has an ionization enthalpy Δ*_b_H_buffer_* equal to zero. However, it is not equal to the intrinsic binding enthalpy.

The intrinsic (buffer-independent) enthalpy of ICPD compound binding was estimated from the relationship:
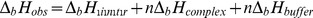
(4)


### Protein Denaturation Experiments by the Thermal Shift Assay (TSA)

The TSA was performed using Corbett Rotor-Gene 6000 (QIAGEN Rotor-Gene Q) spectrofluorimeter. The prepared protein concentration was usually 5 µM and the ligand concentrations varied from 0 to 200 µM. Buffer containing 50 mM sodium phosphate and 100 mM sodium chloride was usually used for TSA experiments. Up to 2% (v/v) of DMSO was added to the solution in each measurement. Reaction volume was usually 10 µl. Unfolding of the protein was monitored by measuring the fluorescence of the 1,8-anilinonaphthalene sulfonate (ANS), at 50–100 µM. The samples were heated at a rate of 1 °C/min. The samples were excited with 365±5 nm UV light and ANS fluorescence emission was registered at 460±5 nm light. Protein melting temperatures were determined by fitting the protein melting curves according to Eq. (5):
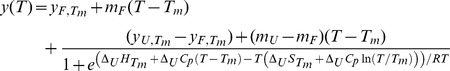
(5)


*y*(*T*) is the calculated fluorescence as a function of temperature; 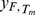 is the fluorescence of the probe bound to folded native protein before the transition at *T_m_*; 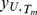 is the fluorescence of the probe bound to the unfolded protein after the unfolding transition at *T_m_*; *m_F_* is the slope of the fluorescence dependence on temperature when the probe is bound to the native protein; *m_U_* is the slope of the fluorescence dependence on temperature when the probe is bound to the unfolded protein; 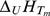 is the enthalpy of protein unfolding at *T_m_*; 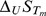 is the entropy of protein unfolding at *T_m_*; 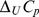 is the heat capacity of protein unfolding and is assumed to be temperature-independent over the temperature range studied; *R* is the universal gas constant; and *T* is the absolute temperature (Kelvin).

The curves in Figure 4 were fit according to Eq. (6), yielding the binding constants for studied inhibitors.
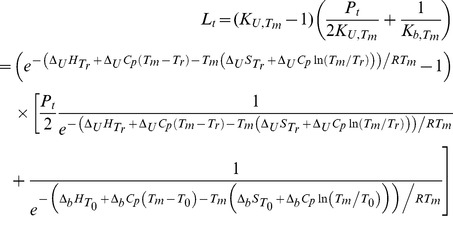
(6)


*L_t_* is the total concentration of added ligand, 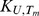 is the protein unfolding equilibrium constant at *T_m_*; *P_t_* is the total protein concentration; 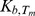 is the ligand binding constant at *T_m_*; 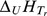 is the enthalpy of protein unfolding at *T_r_*; *T_r_* is the protein melting temperature when no ligand is added; 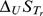 is the entropy of protein unfolding at *T_r_*; 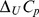 is the heat capacity of protein unfolding and is assumed to be temperature-independent over the temperature range studied; 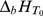 is the enthalpy of ligand binding at *T*_0_; *T*_0_ is the temperature at which the binding process is studied (usually 37°C); 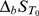 is the entropy of ligand binding at *T*_0_; and 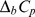 is the heat capacity of ligand binding and is assumed to be temperature-independent over the temperature range studied.

The binding constant at the physiological temperature *T_0_* is determined using Eq. (7):
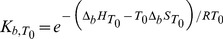
(7)

