# Correction: Thermodynamics of Aryl-Dihydroxyphenyl-Thiadiazole Binding to Human Hsp90

**DOI:** 10.1371/annotation/fb1b3941-789b-4d6f-8463-19a877124f78

**Published:** 2012-06-07

**Authors:** Egidijus Kazlauskas, Vilma Petrikaitė, Vilma Michailovienė, Jurgita Revuckienė, Jurgita Matulienė, Leonas Grinius, Daumantas Matulis

The following information was missing from the Funding section: We also acknowledge FP7-REGPOT-2009-1 grant "MoBiLi", agreement no.: 245721 and the COST projects TD0905 and CM0804. 

